# Utilization of Antenatal Care Services in Dalit Communities in Gorkha, Nepal: A Cross-Sectional Study

**DOI:** 10.1155/2018/3467308

**Published:** 2018-11-01

**Authors:** Mamata Sherpa Awasthi, Kiran Raj Awasthi, Harish Singh Thapa, Bhuvan Saud, Sarita Pradhan, Roshani Agrawal Khatry

**Affiliations:** ^1^Department of Nursing, Janamaitri Foundation Institute of Health Sciences, Hattiban, Lalitpur, Nepal; ^2^Save the Children, Malaria Program, Nepal; ^3^Department of Pharmacy, Janamaitri Foundation Institute of Health Sciences, Hattiban, Lalitpur, Nepal; ^4^Department of Medical Laboratory Technology, Janamaitri Foundation Institute of Health Sciences, Hattiban, Lalitpur, Nepal; ^5^Department of Nursing, Lalitpur Nursing Campus, Sanepa, Lalitpur, Nepal

## Abstract

**Background and Objective:**

Antenatal care (ANC) is one of the main components of maternal health. Utilization of safe motherhood is deprived in women who belong to low-caste groups like Dalit of Nepal. Low socioeconomic status, poor knowledge and awareness on obstetric complications, lack of decision-making autonomy, and limited health care options lead to underutilization of existing maternal health care service. The aim of this study was to ascertain the utilization of antenatal care services in terms of ANC visits with health personnel, receiving recommended period of iron tablets, consumption of antihelminthes and number of Tetanus Toxoid (TT) vaccines taken among child bearing women in Dalit community.

**Materials and Methods:**

Descriptive cross-sectional research design was used to conduct the study of 150 child bearing women of reproductive age (15-49 years) having at least one child up to three years of age in a Dalit community of Gorkha from March 2015 to March 2016. The data was collected from each mother by conducting face to face interview with each household by using a questionnaire.

**Result:**

The study revealed that mean age at marriage of respondents was 17.7 years and mean age at first pregnancy was 18 years. 44.6% of respondents experienced complication during last pregnancy, labour, and postpartum period in their last pregnancy. 59.3% of respondents stated that neighbors, relatives, and traditional healers were the best first contact person during health problem of women. 76.0% of respondents had attended antepartum visit during their last pregnancy whereas 24.0% of respondents did not attend any antepartum clinic. 68.3% of the mothers had consumed Iron/Folates within 45 days after delivery. Only 30.0% of respondents received antihelminthes (albendazole) while 70.0% of respondents had received TT Vaccines during their last pregnancy. Age, type of family, and education of the mothers were significantly associated with utilization of antenatal care services.

**Conclusion:**

Even though there is reasonable good utilization rate of antenatal service, the study revealed that low education and awareness among mothers, low socioeconomic condition, early marriage and pregnancy, inappropriate antenatal health check-up, and cultural taboos were significant factors affecting the satisfactory utilization of services among the Dalit community. Hence, there is a need to emphasize on raising awareness of Dalit mothers for receiving available prenatal services.

## 1. Introduction

Maternal mortality is one of the major causes of death among women of reproductive age in developing countries. The annual number of maternal deaths was estimated 303,000 in 2015. Globally, maternal death accounted to be approximately 99% deaths in developing regions [1]. Despite Antenatal care (ANC) being one of the four major initiatives of the Safe Motherhood Initiative, its relative contribution to improving the maternal health morbidity and mortality still is uncertain and debatable. Nonetheless some of the ANC procedures have been found to be beneficial [2]. World Health Organization (WHO) recommends a four-visit ANC schedule for low risk pregnancies [3]. During these visits, the components of ANC suggested in Nepal include iron supplementation, blood and urine tests, at least two Tetanus Toxoid (TT) injections, blood pressure measurement, intestinal parasite drugs, and health education related to pregnancy and detection of the problems that make the pregnancy high risk one [4, 5]. It is difficult to predict which expectant mother will develop pregnancy related complications that is why it is essential that all pregnant women must have an access to high quality obstetric care throughout their pregnancies. Maternal complications and poor perinatal outcome are usually associated with other factors like nonutilization of antenatal and delivery care services and poor socioeconomic condition of the family. Unwanted outcomes are seen in those mothers who have not done ANC registration compared to registered one [6].

In Nepal, utilization of maternal health services depends on the Socio Economic Status (SES) of women. Higher SES women in terms of education level, wealth and urban residence utilize better health care services including maternity care [7]. Dalits are defined as untouchables and marginalized groups within the Hindu caste system. Often the social, economic, and health status and political conditions among these population are lowest compared to other groups in Nepal [8]. Women from Dalit community suffer from not only discrimination based on their gender but also caste identity and consequent economic deprivation [9]. Dalit women face numerous barriers in accessing maternal healthcare services due to their lower status in the society and, in Nepal, women with disabilities and women from Dalit caste groups are with low rates of maternal health service utilization [10, 11].

A study done in Bara district in the same community found that 41.6% of mothers did not receive any antenatal checkup while only 28.0% completed all four ANC visits, more than 55.0% had not taken vaccine, and 48.3% had not taken folic acid during pregnancy period [12]. Based on the Dalit women's status in the country, it is therefore necessary to identify the gaps in the utilization of antenatal health care services so that the condition of Dalit women, particularly their awareness and utilization of antenatal health can be improved. Therefore, the present study will help to investigate the status of ANC services, i.e., ANC visits with health personnel, receiving days of iron tablets and number of TT vaccines taken during last pregnancy in Dalit community in rural area health care facilities.

## 2. Materials and Methods

Quantitative descriptive cross-sectional design was adopted to find out the utilization of antenatal care services among child bearing women of reproductive age (15-49 years) having at least one child up to three years of age in a Dalit community between March 2015 to March 2016 in wards one, two, and three in Gorkha municipality, a hilly district in central Nepal. A list of households having child bearing women with child up to 3 years from these wards where predominantly Dalit communities resided was obtained from Female Community Health Volunteers (FCHV). A total number of 150 child bearing women having at least one child up to 3 years of age were considered for this research. Pretesting of the instrument was done in 16 (10%) of child bearing women having a child up to 3 years in ward number 8 Tanchintar of Godawari where Dalit community were predominant. Permission from the concerned authorities was obtained by submitting a written request letter to the local Village development committee. Selected child bearing women having child up to 3 years were interviewed from each household. The confidentiality of respondent was maintained through code numbers in questionnaire. Each respondent was informed that she had right to withdraw from the study at any time without prejudice to her care. An informal verbal consent was obtained from each of them prior to conducting the face to face interview for data collection. The duration of interview was about 40-50 minutes which was mentioned prior to conducting the interview.

## 3. Data Analysis

The collected data was analyzed by using Statistical Package for Social Science (SPSS) 20 version. Descriptive statistics such as frequency, percentage, mean, standard deviation, and inferential statistics (chi-square test) was used to measure the association between different variables and utilization of antenatal care services in the Dalit community.

## 4. Results

Table 1 shows the sociodemographic characteristics of the women. Out of the total women in the study 51.4% of respondents were of age group of less than or equal to 25 years while 73.3% of the respondents had married at an age of less than or equal to 18 years while the mean age of marriage was 17.7. A total of 84.7% of the respondents had become pregnant for the first time at an age of less than or equal to 20 years with the mean age of pregnancy being 18 years. Out of these mothers 7.3% of them had given birth more than four times. It was found that 75.3% of respondents belonged to a nuclear family (not living with their inlaws) while the rest of them live in joint families (couple living with their inlaws). The results showed that 68% of the decision makers in the family were husbands. In this study all of the respondents were Hindus. Primary level of education was completed by only 44.7% of the respondents where as 26.6% of respondents were illiterate. Nearly half of the mothers were housewives and worked at home.

As illustrated in Table 2, 76.0% of respondents had attended an ANC clinic out of which a further 72.8% of had undergone four or more ANC visits. It was observed that 51.8% of the respondents had their first ANC visit in the fourth or later months of pregnancy. All of the respondents who visited hospital for antepartum care attended a government hospital. Table 2 depicts that 24.0% of respondents did not attend any antepartum care. The main reasons for not attending an antepartum clinic by 38.9% of mothers were due to inadequate knowledge regarding obstetric complication and availability of free safe motherhood services across the public hospitals. Furthermore, 27.7% of the respondents indicated that that they did not face any problem/complication during their pregnancy; therefore did not feel the need of any ANC care, while the remaining 16.7% cited lack of time due to household choirs, field work, and traditional views of their inlaws that pregnancy based on their personal experiences as the main reasons for not undergoing the predelivery checkups.

As shown in Table 3, 82.0% of respondents had consumed Iron/Folates out of which 68.3% of them had consumed Iron/Folates up to 45 days postdelivery. Only 30.0% of respondents received antihelminthes (albendazole). The reason for not taking antihelminthes in 85.9% of the mothers was due to the fact that they had not been prescribed by the health facility. This could be because the hospital health care providers were not aware of prescribing antihelminthes during pregnancy or may have been due to stock out of the medicines in their facility. In addition to this, it was observed that a majority of respondents had attended antepartum care in a nearby government hospital. A total of 70% mothers had received TT Vaccines and among them 65.7% received both the recommended doses during the period of their pregnancy.

Table 4 indicates that around two third of respondents utilized antenatal care services well whereas 39.3% of respondents had poor utilization of antenatal care services.

The association between sociodemographic variables and utilization of antenatal care services has been illustrated in Table 5. It was observed that 88.3% of the respondents aged less than 25 years utilized antenatal care services more suggestive of a statistically significant association between age of mother and utilization of antenatal care services. Furthermore, the results also illustrated that 76.4% of literate mothers underwent safe motherhood practices. This high percentage indicates a significant relationship between educational status and utilization of antenatal care services at 95% level of confidence (p < 0.05). Mothers from nuclear families were strongly associated with utilization of antenatal care services 95% level of confidence (p < 0.05) compared to those that live in joint families. Age at marriage and age at first pregnancy of respondents were not found to be statistically significant to utilization of the Safe Motherhood Practices.

## 5. Discussion

The current study showed that the mean age of the mothers was 25.8 (SD, 3.6). A total of 73.3% of respondents got married at an age of less than or equal to 18 years with a mean age of marriage among all respondents at 17.7 years (SD, 1.7). It was noted that 84.7% of the respondents became pregnant before they were 20 years which is similar to the findings of the study done at Bara district of Nepal among a similar Dalit community where 53.3% were married between the age of 15 and 19 years [12]. The results of another study conducted among similar group in Rupandehi of Nepal also showed early pregnancy among 72.55 of the women, which further adds weight to the fact that women from Dalit castes are predisposed to a higher risk of obstetric complications including higher mortality rates [13, 14]. This result pinpoints the fact that majority of women from the lower castes especially the Dalits get married before the legal age of marriage (i.e.18 years) and become pregnant before the age of reproductive maturity. This could be attributed to poor economic status and strong sociocultural beliefs instilled within the community. Early marriage and childbearing age plays a significant role in reproductive health and utilization of maternal health care practices. Studies have proven that pregnancy at an early age could lead to miscarriage and even bring about unwanted complications during and after pregnancy as a woman at such an early age would not be having a mature body structure [15].

With regards to education level of the respondents, secondary level of education was completed by only 24.0% of the respondents whereas 26.7% of respondents were illiterate. Educated mothers are more aware of their health and development of the family. The literacy rate of Dalit females is quite low compared to the national female literacy rate which is 57.4% according to 2011 census [16]. It was noted that almost half of the respondents 49.3% were housewives. The husbands were the main decision makers in the household (68.0%), followed by the father in laws 17.3% whereas only a few number of respondents took their own decisions on healthcare. Women have little preference in the family; hence they have to rely on their husband and family members (mostly inlaws) to take any decision. It is even more important for the women to make their own choices and decisions based on the adequate information of the services they use as per their personal, family, and social needs. Studies have revealed that both economic status and social dynamics regarding distribution of power between spouses have an influence on the use of maternal health services [17]. In our study, around 75.0% of the respondents belonged to a joint family. This was slightly higher compared to a similar study done in the Mid-Western region of the country where 58.2% of the mothers belonged to a joint family [18]. It is often seen that a good marital relationship between spouses exists when they live in nuclear families and for those women living in joint families better relation with their mother in laws results in better utilization rates of the ANC services during pregnancy [19].

In this study, 40.7% of the respondents stated that the first person of contact during pregnancy related issues, childbirth and postpartum period were health workers while 59.3% of respondents identified others such as neighbors, relatives and traditional healers as their immediate contact with the preference more on the later. This help seeking behavior could be attributed to various factors including their availability, cost effectiveness, and deep rooted traditional and cultural values in the society. In rural areas, people put their faith and prefer to seek care from traditional healers, a social behavior that has been passed down to them across generations from their ancestors [20].

With regards to antenatal health check-up during their latest pregnancy, 76.0% of respondents had attended antepartum visit in their last pregnancy, a figure that is higher than national statistics (NDHS, 2011) average of 58.3% [21]. Similar results were seen in studies from Lalitpur and Ilam districts of Nepal where majority of the women attended ANC in the last pregnancy. Around 79.0% of the women in Lalitpur and 95.0% in Ilam underwent ANC visits during the last episode of pregnancy [22, 23]. Despite the provision of free maternal health care services provided by Government of Nepal across all government hospitals, 24.0% of respondents did not attend any antepartum care. This finding is similar to the study done in hilly area of Nepal where 21.1% did not attend any ANC visit [22]. The reason for not attending antepartum clinic was due to the lack of knowledge regarding obstetric complication and free delivery services among 38.9% of the respondents followed by 27.7% of the women experiencing no complications during their pregnancy. Similarly, 16.7% cited lack of time due to household choirs and incumbent traditional views within the family as the reason for the low service utilization. During the interview process the several factors that led to underutilization of ANC services included a lower SES of the women in the community as well as the persistent traditional belief that pregnancy was a natural phenomenon which did not require additional health checkup like their in-laws during their pregnancies. It was found that the mothers-in-laws were not supportive and did not encourage the ANC checkups as the pregnant women were expected to carry out the daily household choirs.

Despite the Government of Nepal launching a Safe Motherhood Initiative Program to promote utilization of institutional delivery services which includes free delivery care with 4 ANC visit incentives for women, complete ANC visit is still quite low. Our study revealed that among 114 respondents who attended ANC visits, 7.0% of them visited ANC once, 3.5% visited twice, 16.7% visited thrice, and the remaining majority 72.8% percent of them completed all four ANC visits. This finding is consistent with the study done in Nepal where that 70.0% of mothers underwent four antenatal visits, 14.0% did 3 antenatal visits 13.7% with 2 antenatal visits, and 2.1% had a single antenatal visit [24]. Furthermore, among the 114 respondents who attended first ANC visit at a health facility, 51.9% of the respondents had their first antenatal visit at 4 months or later. Similarly, 27.1% of respondents had an antepartum visit in the third month of pregnancy while 21.0% of respondents had their first ANC visit during the second month of pregnancy. Among them who had done their ANC visit, all of the respondent had visited a government hospital. In contrast to this, present study revealed that 56.0% were registered for ANC in the first trimester of pregnancy, whereas only 1.0% were registered in the third trimester. They were registered at different level of health services, i.e., 1.8% in subhealth posts, 28.0% in PHCs, 34.3% in a Zonal hospital, and 35.9% in private nursing homes [24]. This variation must have resulted from the existing good and free maternal and child health services developed for pregnant mother across all public health facilities in the country.

Among the participants, it was observed that 82.0% of the mothers had consumed Iron/Foliates out of which a further 68.3% of them had consumed the tablets up to 45 days after delivery. The study also revealed that only one third of respondents received an anti-helminths (albendazole). Around 70.0% of respondents had received TT Vaccines out of which only 65.7% of respondents received both doses with the remaining mothers either receiving a single or a booster dose of the TT Vaccine during their pregnancy. This finding is lower than National Family Health Survey (NFHS-3) of India average of 73.6% scheduled caste receiving two or more doses and 1.5% receiving a single dose of TT injection during their pregnancy [25]. This finding was similar to another study which showed TT immunization of expecting mothers with 62.3% receiving 2 doses and 37.7% receiving a single or booster dose [24]. In this study, among 92 respondents that did not receive anti-helminths, 85.9% stated that they had not been prescribed the same by the health person. This could be due to the lack of adequate supply of free Anti-helminths medicine across public health facilities or also could be attributed to the lack of knowledge on prescribing the anti-helminths among the health workers.

Our study revealed that 60.7% of the mothers utilized the existing ANC care services well whereas the remaining 39.3% of the respondents showed poor utilization of antenatal care services. While assessing the sociodemographic determinants, it was observed that age (p=0.001), education levels of respondents (p=0.001), and type of family of respondents (p =0.001) were strongly associated with utilization of the ANC services; however, age at marriage (p=0.302) and age at first pregnancy (p=0.342) did not affect the utilization of ANC services among women. This result illustrates that 76.4% of educated women, 88.3% of women below 25 years of age, and 70.8% of them from nuclear families utilized antenatal health care services more than respondents who were illiterate and above 25 years of age and lived in a joint family. This could be attributed to lack of time from household activities and pressure from in laws to the mothers of Dalit communities. Younger mothers (less than age 20) are more likely to receive antenatal care from a skilled provider than older mothers (age 35-49) [21]. Other studies have also reflected how educated mothers often seek more ANC services than those that are not literate [26, 27]. Therefore the 2001 Nepal Demographic and Health Survey study in its recommendation outlined the emphasis on better maternal education to help improve the greater utilization of maternal health care services among women in Nepal [28]. It is important to focus on education of the women from the lower caste groups such as Dalits as the dropout rates in these communities is very high [29, 30]. It is also necessary to provide more emphasis to uplift the awareness levels of Dalit mothers for receiving available prenatal services [31].

Several factors such as lower SES and low level of awareness on ANC are inversely related to overall quality of health among women including safe motherhood practices especially among marginalized Dalit population [12].

## 6. Conclusion

Antenatal care is an essential component of safe motherhood. The study revealed that the overall utilization of antenatal health services was good as almost two third of the mothers utilize it, while more than one third of them had poor utilization of antenatal care services. Regardless of free safe motherhood services in government hospital and high utilization rate of maternal health care services utilization of all four ANC visits, consumption of iron tablet, anti-helminthes and administration of TT vaccine during their last pregnancy was considerably low among the women in the Dalit community. The study also revealed that educational status of mothers, age and type of family played a significant role in underutilization of ANC services among the women from lower castes. The study identifies the need to hence look at the significant factors associated with utilization of the available free safe motherhood services across all public health facilities especially among Dalit communities and to address this prioritized intensive awareness programs and behavioral change interventions on maternal health and socioeconomical development for women in Dalit communities should be planned. Community engagement and social awareness could play a very crucial role to help promote maternal health thereby impacting the overall maternal health of these women in the future. The policy-makers should also advocate for prioritizing accessibility and such awareness raising activities targeting underserved communities such as the Dalit women residing in rural hard to reach areas.

## Figures and Tables

**Table 1:** Respondents' characteristics.

**Items**	**Frequency**	**Percentage**
**Age (in Years)**		
<=25	77	51.4
26-30	59	39.3
31=>	14	9.3
Mean ±SD =	25.8±3.6	
**Age at Marriage (in Years)**		
<=18	110	73.3
19=+	40	26.7
Mean ±SD =	17.7±1.7	
**Age at First Pregnancy(in Years)**		
<=20	127	84.7
20=+	23	15.3
Mean ±SD =	18±1.7	
**Number of Pregnancies**		
One Time	39	26.0
Two Times	51	34.0
Three Times	49	32.7
Four or more Times	11	7.3
**Type of family**		
Nuclear Family	113	75.3
Joint Family	37	24.7
**Decision Maker of the family**		
**Husband**		
Father-in-Law	26	17.4
Mother-in-Law	11	7.3
Husband	102	68.0
Self	11	7.3
**Religion**		
Hindu	150	100.0
**Education level**		
Illiterate	40	26.6
Primary level	67	44.7
Secondary level	36	24.0
Higher secondary level	7	4.7
**Occupation**		
House-maker	74	49.4
Business	8	5.3
Service	7	4.7
Daily wages	53	35.3
Agriculture	8	5.3

**Table 2:** ANC visits for pregnancy checkups.

**Item**	**Frequency**	**Percentage (**%**)**
**ANC Visits**		
Yes	114	76.0
No	36	24.0
**Number of ANC visit during last pregnancy(n=114)**		
Once	8	7.0
Two times	4	3.5
Three times	19	16.7
Four times or more	83	72.8
**Month of ANC visit for first time(n=114)**		
2nd month of pregnancy	24	21.1
3rd month of pregnancy	31	27.1
4th and later month of pregnancy	59	51.8
**Place of antenatal visit for check up during last pregnancy(n=114)**		
Government	114	100.0
**If not visited, reason for not visit health centre for antepartum care(n=36)**		
Lack of Knowledge	14	38.9
Lack of time	6	16.7
Traditional View of in laws	6	16.7
Not having any problem during pregnancy	10	27.7

**Table 3:** Respondents' utilization of medicines during antepartum period.

**Items**	**Frequency**	**Percentage (**%**)**
**Consumption of Iron/Foliate**		
Yes	123	82.0
No	27	18.0
**If yes, duration of iron/foliate tablets consumed(n=123)**		
until delivery	39	31.7
up to 45days after delivery	84	68.3
**Intake of anti-helminthes (n=150)**		
Yes	45	30.0%
No	92	61.3%
Don't remember(Unsure)	13	8.7%
**Reason for not taking Anti-helminthes (n=92)**		
Not prescribed	79	85.9%
No Antepartum Visit	13	14.1%
**TT Vaccine (n=150)**		
Yes	105	70.0
No	45	30.0
**Number of TT vaccine **		
**Received (n=105)**		
One dose or booster	36	34.3%
Two doses	69	65.7%

**Table 4:** Overall utilization of antenatal care services.

**Item**	**Frequency**	**Percentage**
**Utilization of Antenatal Care Services**		
Poor Utilization (up to 50%)	59	39.3
Good Utilization (above 50%)	101	60.7

**Table 5:** Association between sociodemographic variables and utilization of antenatal care services.

**Utilization of Antenatal Care Services**					
**Demographic** **Variables**	**Poor** **Utilization**	**Good** **Utilization**	**Total**	**χ** ^2^	**P** **Value**
**Age**					
Up to 25 years	9(11.7%)	68(88.3%)	77	50.674	**0.001**
Above 25years	50(68.5%)	23(31.5%)	73		
**Educational status**					
Illiterate	33(82.5%)	7(17.5%)	40	42.594	**0.001**
Literate	26(23.6%)	84(76.4%)	110		
**Age at Marriage**					
Up to 18 Years	46(41.8%)	64(58.2%)	110	1.067	0.302
Above 18 Years	13(32.5%)	27(67.5%)	40		
**Age at first pregnancy**					
Up to 19 years	52(40.9%)	75(59.1%)	127	0.901	0.342
Above 19 years	7(30.4%)	16(69.5%)	23		
**Type of Family**					
Nuclear	33(29.2%)	80(70.8%)	113	19.700	**0.001**
Joint	26(70.3%)	11(29.7%)	37		

## Data Availability

The authors agree that anyone interested in accessing the raw data from the research can be provided upon request to the authors in the email address oralhealth.hdsn@gmail.com. The data will be provided to anyone based on the two conditions: upon use of the data the authors should be acknowledged and also the paper needs to be cited. An agreement in the aforementioned situation will be done if agreed upon.

## List of Abbreviations

ANC: Antenatal care
TT: Tetanus Toxoid
SES: Socio Economic Status
SPSS: Statistical Package for Social Science
FCHV: Female Community Health Volunteers
WHO: World Health Organization.
